# Supplementary material for: Beneficial effects of resistance training on both mild and severe mouse dystrophic muscle function as a preclinical option for Duchenne muscular dystrophy
Source: PLoS One. 2024 Mar 8;19(3):e0295700. doi: 10.1371/journal.pone.0295700 (PMC10923407; doi:10.1371/journal.pone.0295700)
Supplement: S1 Table — (DOCX) [file pone.0295700.s001.docx]

S1 Table. Correlations.

---------------------------------------------------------------------------------------------------------------------------

R p value

---------------------------------------------------------------------------------------------------------------------------

Correlations between the pourcentage of remaining force following the 9th Lengthening contraction and muscle weight

Experiment 1 0.544 p = 0.0023

Experiment 2 0.417 p =0.022

Experiment 3 0.594 p = 0.0005

Correlation between the ratio pAkt/Akt and muscle weight (Experiment 3)

0.743 p = 0.0206

---------------------------------------------------------------------------------------------------------------------------
