# Supplementary material for: Beneficial effects of resistance training on both mild and severe mouse dystrophic muscle function as a preclinical option for Duchenne muscular dystrophy
Source: PLoS One. 2024 Mar 8;19(3):e0295700. doi: 10.1371/journal.pone.0295700 (PMC10923407; doi:10.1371/journal.pone.0295700)
Supplement: S1 File — Plantaris mdx, Force (experiment 1). (PDF) [file pone.0295700.s002.pdf]

	P0	Excentric											
Plantaris	0	%	3	%	6	%	9	%		weight	sP0	body weight	TA (mg)
mdx	(g)									mg	g/mg	g	
14	55,5	100,0	42,4	76,4	18,8	33,9	10,4	18,7		21,4	2,6	28,4	111
	27,8	100,0	22,1	79,5	18,8	67,6	15,4	55,4		21,6	1,3		111,5
16(tube number 15)	20,4	100,0	5,7	27,9	3,6	17,6	3,0	14,7		15,9	1,3	24,4	78
	30,9	100,0	21,5	69,6	13,4	43,4	10,2	33,0		18,8	1,6		80,1
17	33,3	100,0	28,9	86,8	22,6	67,9	17,9	53,8		25,5	1,3	33,6	80,5
	40,1	100,0	33,8	84,3	17,2	42,9	6,0	15,0		28	1,4		91,3
20	32,1	100,0	13,9	43,3	7,4	23,1	3,3	10,3		21,6	1,5	29,4	102,4
	32,8	100,0	18,3	55,8	12,7	38,7	9,6	29,3		23,9	1,4		92,4
22	29,0	100,0	13,8	47,6	6,6	22,8	4,0	13,8		24,1	1,2	30,9	84,4
	41,7	100,0	18,1	43,4	8,0	19,2	8,0	19,2		24,3	1,7		94,3
24	31,7	100,0	21,5	67,8	14,3	45,1	12,4	39,1		18,8	1,7	26	76,5
	18,6	100,0	12,8	68,8	5,7	30,6	6,7	36,0		20,1	0,9		
mean	32,8	100,0	21,1	62,6	12,4	37,7	8,9	28,2	##	22,0	1,5	28,8	91,1
SE	9,8	0,0	10,0	18,7	6,1	17,0	4,7	15,5	##	3,4	0,4	3,3	12,7
SEM	2,8	0,0	2,9	5,4	1,8	4,9	1,4	4,5	##	1,0	0,1	1,4	3,8
mdx+OVL													
1	60,7									51,7	1,2	27	78
										31,8			74,9
3	105,0	100,0	82,7	78,8	74,3	70,8	66,6	63,4		53,5	2,0	31,9	74,2
	73,3	100,0	51,1	69,7	40,2	54,8	34,8	47,5		38	1,9		85,6
5	66,9	100,0	29,7	44,4	27,3	40,8	24,4	36,5		20,2	3,3	27	76,1
	82,0	100,0	48,1	58,7	42,9	52,3	35,0	42,7		17,8	4,6		75
7	64,7	100,0	54,3	83,9	44,6	68,9	40,0	61,8		25,9	2,5	27,5	83,1
	43,0	100,0	35,0	81,4	25,0	58,1	23,5	54,7		22,6	1,9		80,4
9	62,5	100,0	53,6	85,8	48,0	76,8	39,9	63,8		23,6	2,6	26	84,4
	65,0	100,0	49,1	75,5	41,8	64,3	37,9	58,3		26,4	2,5		76,9
11	91,9	100,0	77,4	84,2	65,5	71,3	59,0	64,2		34,8	2,6	30,4	104,7
	80,9	100,0	62,2	76,9	53,7	66,4	49,4	61,1		33,2	2,4		91,5
mean	72,4	100,0	54,3	73,9	46,3	62,5	41,1	55,4	##	31,6	2,5	28,3	82,1
SE	16,9	0,0	16,5	13,2	15,3	10,9	13,8	9,9	##	11,5	0,9	2,3	8,9
SEM	5,1	0,0	5,2	4,2	4,8	3,4	4,4	3,1	##	3,3	0,3	0,9	2,6
student vs mdx	0,00	#DIV/0!		0,12		0,00		0,00	##	0,01	0,00	0,78	0,06
%mdx	220,4									143,8	167,7	98,3	90,1
mdx+OVL+CSA													
13	49,2	100,0	34,1	69,3	30,9	62,8	30,6	62,2		31,4	1,6	28,7	84,5
	41,0	100,0	30,9	75,4						22,7	1,8		84,1
15(tube number 16)	72,0	100,0	58,6	81,4	53,4	74,2	47,1	65,4		34,3	2,1	28,3	82,3
	60,9	100,0	53,6	88,0	48,0	78,8	43,8	71,9		32,1	1,9		73,1
18	56,4	100,0	47,7	84,6	41,3	73,2	38,2	67,7		31,4	1,8	27,8	51,5
	42,2	100,0	38,1	90,3	32,0	75,8	31,4	74,4		32,7	1,3		57,2
19										29,4		28,6	64,4
	94,9	100,0	63,3	66,7	51,3	54,1	46,6	49,1					72,2
21										29,8		24,3	60,5
										21,5			58
23	105,1	100,0	71,0	67,6	58,0	55,2	50,7	48,2		30,6	3,4	23,7	42,8
	50,3	100,0	34,0	67,6	30,2	60,0	27,1	53,9		24,8	2,0		50,7
mean	63,6	100,0	47,9	76,8	43,1	66,8	39,4	61,6	##	29,2	2,0	26,9	65,1
SE	22,9	0,0	14,5	9,5	11,1	9,9	8,9	10,1	##	4,2	0,6	2,3	14,1
SEM	7,6	0,0	4,8	3,2	3,9	3,5	3,1	3,6	##	1,3	0,2	0,9	4,1
student vs mdx	0,00	#DIV/0!		0,053		0,00		0,00	##	0,00	0,05	0,28	0,00
%mdx	193,6								##	132,5	133,1	93,5	71,4
student vs mdx+OVL	0,34	#DIV/0!	0,38	0,60	0,63	0,40	0,78	0,21	##	0,51	0,18	0,32	0,00
