# Supplementary material for: Beneficial effects of resistance training on both mild and severe mouse dystrophic muscle function as a preclinical option for Duchenne muscular dystrophy
Source: PLoS One. 2024 Mar 8;19(3):e0295700. doi: 10.1371/journal.pone.0295700 (PMC10923407; doi:10.1371/journal.pone.0295700)
Supplement: S2 File — Lat Gast Mdx, force (experiment 2). (PDF) [file pone.0295700.s003.pdf]

LG	P0		Excentric										
MDX + OVL	0	%	3	%	6	%	9	%		Muscle weight	sP0	Muscle/body	Body weight
	(g)									mg	g/mg	mg/g	g
1	127,5	100,0	94,3	74,0	72,0	56,5	68,9	54,0		104,4	1,2	3,5	30,1
3	84,4	100,0	70,9	84,0	37,0	43,8	33,8	40,0		105,1	0,8	3,8	27,7
	93,9	100,0	46,7	49,7	36,3	38,7	31,8	33,9		77,7	1,2	2,8	27,7
4	174,7	100,0	86,3	49,4	47,5	27,2	42,8	24,5		133,6	1,3	3,8	35,0
	211,8	100,0	79,9	37,7	55,7	26,3	47,5	22,4		131,7	1,6	3,8	35,0
6	80,1	100,0	49,9	62,3	40,7	50,8	38,8	48,4		126,4	0,6	3,8	33,4
	124,8	100,0	88,5	70,9	72,6	58,2	65,1	52,2		124,0	1,0	3,7	33,4
7	114,8	100,0	79,3	69,1	61,0	53,1	55,3	48,2		93,8	1,2	3,3	28,2
	133,0	100,0	59,6	44,8	44,6	33,5	40,1	30,2		98,0	1,4	3,5	28,2
8	96,8	100,0	51,5	53,2	32,6	33,7	29,2	30,2		115,3	0,8	4,3	26,9
	104,9	100,0	46,2	44,0	39,4	37,6	34,8	33,2		98,3	1,1	3,7	26,9
13	180,7	100,0	115,6	64,0	83,8	46,4	71,4	39,5		143,8	1,3	4,2	34,3
	150,3	100,0	118,6	78,9	88,4	58,8	75,2	50,0		115,4	1,3	3,4	34,3
14	146,6	100,0	73,5	50,1	62,1	42,4	73,6	50,2		112,2	1,3	3,0	37,6
										100,0	0,0	2,7	37,6
16	113,4	100,0	69,3	61,1	48,7	42,9	44,7	39,4		149,4	0,8	4,3	34,7
	161,4	100,0	96,0	59,5	70,4	43,6	65,8	40,8		128,4	1,3	3,7	34,7
mean	131,2	100,0	76,6	59,5	55,8	43,3	51,2	39,8		115,1	1,1	3,6	32,1
SE	39,1	0,0	23,8	14,4	18,4	11,1	17,1	10,8		19,2	0,4	0,5	3,8
SEM	12,3	0,0	7,5	4,5	5,8	3,5	5,4	3,4		6,1	0,1	0,1	1,2
student vs MDX	0,04	###	0,02	0,14	0,00	0,00	0,00	0,00	#	0,00	0,38	0,00	0,63
%MDX	122,7									126,2	91,5	128,4	98,3
MDX													
2	104,1	100,0	42,6	40,9	24,5	23,5	20,8	20,0		92,1	1,1	2,8	32,9
	111,4	100,0	41,9	37,6	23,9	21,5	26,4	23,7		92,6	1,2	2,8	32,9
5	132,9	100,0	75,3	56,7	54,1	40,7	46,4	34,9		110,5	1,2	3,0	37,3
	99,9	100,0	69,2	69,3	48,8	48,8	36,5	36,5		93,2	1,1	2,5	37,3
9	113,4	100,0	78,1	68,9	52,5	46,3	42,1	37,1		92,6	1,2	3,2	29,0
	51,6	100,0	22,5	43,6	13,8	26,7	13,6	26,4		83,6	0,6	2,9	29,0
10	96,7	100,0	50,0	51,7	29,1	30,1	19,9	20,6		94,2	1,0	2,8	33,5
	135,3	100,0	79,0	58,4	34,4	25,4	23,9	17,7		88,0	1,5	2,6	33,5
11	138,6	100,0	79,6	57,4	34,7	25,0	24,6	17,7		100,7	1,4	3,1	32,5
	98,8	100,0	52,2	52,8	26,9	27,2	17,4	17,6		82,5	1,2	2,5	32,5
12	94,8	100,0	57,6	60,8	33,5	35,3	27,8	29,3		87,7	1,1	2,7	32,3
	97,3	100,0	28,8	29,6	13,2	13,6	22,3	22,9		82,3	1,2	2,5	32,3
17	109,5	100,0	58,6	53,5	36,6	33,4	30,7	28,0		89,4	1,2	2,9	31,2
	112,3	100,0	61,2	54,5	36,8	32,8	27,0	24,0		88,1	1,3	2,8	31,2
mean	106,9	100,0	56,9	52,5	33,1	30,7	27,1	25,5		91,3	1,2	2,8	32,7
SE	21,7	0,0	18,5	11,3	12,6	9,7	9,2	6,9		7,5	0,2	0,2	2,4
SEM	6,3	0,0	5,3	3,3	3,6	2,8	2,7	2,0		2,2	0,1	0,1	0,7
