# Supplementary material for: Beneficial effects of resistance training on both mild and severe mouse dystrophic muscle function as a preclinical option for Duchenne muscular dystrophy
Source: PLoS One. 2024 Mar 8;19(3):e0295700. doi: 10.1371/journal.pone.0295700 (PMC10923407; doi:10.1371/journal.pone.0295700)
Supplement: S3 File — Plantaris D2-mdx, force (experiment 3). (PDF) [file pone.0295700.s004.pdf]

mdx-dba2 nee juilletaout

OVL

Mice number	Weight(g)	Leg (right D, left G)	Absolute maximal force (g)	Weight of plantaris (mg)	Specific maximal force (g/mg)	Force 4 contractions	% contraction 4/1	Force 7 contractions	% contraction 7/1	Force 10 contractions	% contraction 10/1
		D	17,25	26,80	0,64	14,06	81,51	12,92	74,90	12,23	70,90
2	23,6	G	7,78	25,90	0,30						
		D	28,25	24,00	1,18	19,61	69,42	23,81	84,28	20,25	71,68
3	23,7	G	16,02	25,20	0,64	10,97	68,48	7,98	49,81	7,00	43,70
		D	16,91	31,00	0,55	13,19	78,00	12,56	74,28	12,13	71,73
6	22,6	G		29,30							
		D	17,20	28,60	0,60	12,91	75,06	12,02	69,88	11,34	65,93
7	22,8	G	18,78	27,40	0,69	14,64	77,96	13,34	71,03	12,23	65,12
		D	19,75	27,20	0,73	19,31	97,77	17,30	87,59	13,98	70,78
9	21	G	20,73	23,40	0,89	17,20	82,97	14,84	71,59	13,92	67,15
		D	17,42	21,40	0,81	11,56	66,36	10,13	58,15	9,20	52,81
11	22,5	G	10,31	24,70	0,42						
		D	17,63	23,10	0,76	13,75	77,99	12,95	73,45	11,95	67,78
13	19,9	G	15,97	21,90	0,73	13,63	85,35	11,03	69,07	10,17	63,68
		D	17,73	22,10	0,80	12,53	70,67	11,31	63,79	10,77	60,74
14	22,4	G	25,91	23,90	1,08	22,69	87,57	20,50	79,12	19,41	74,91
		D		11,70							
16	23,4	G	29,34	18,90	2,51	26,97	91,92	21,34	72,73	18,44	62,85
		D	34,13	21,20	1,81	30,75	90,10	30,66	89,83	29,38	86,08
18	23,4	G	21,11	24,60	1,00	16,80	79,58	14,66	69,45	12,91	61,16
Moyenne			19,57	24,12	0,90	16,91	80,04	15,46	72,44	14,08	66,06
student vs non ovl			0,00	0,00	0,39	0,00	0,00	0,00	0,00	0,00	0,00

Non-OVL

Mice number	Weight (g)	Leg (right D, left G)	Absolute maximal force (g)	Weight of plantaris (mg)	Specific maximal force (g/mg)	Force 4 contractions	% contraction 4/1	Force 7 contractions	% contraction 7/1	Force 10 contractions	% contraction 10/1
		D	11,40	21,90	0,52	7,86	68,95	6,13	53,77	5,66	49,65
1	24,4	G	10,84	13,90	0,78	5,63	51,94	4,84	44,65	4,36	40,22
		D	18,66	14,40	1,30	13,89	74,44	9,81	52,57	8,17	43,78
4	22,2	G	6,09	17,10	0,36	1,92	31,53	0,58	9,52	0,08	1,31
		D	15,95	18,30	0,87	10,95	68,65	5,19	32,54	1,55	9,72
5	24,4	G	10,16	16,10	0,63	8,44	83,07	6,03	59,35	4,91	48,33
		D	11,81	16,90	0,70	9,33	79,00	7,02	59,44	5,98	50,64
8	23,5	G	13,73	12,40	1,11	7,91	57,61	6,41	46,69	4,88	35,54
		D	13,17	14,80	0,89	9,03	68,56	7,41	56,26	5,89	44,72
10	20	G		14,90	0,00						
		D	18,72	20,80	0,90	12,34	65,92	9,86	52,67	8,67	46,31
12	22,8	G	15,53	18,10	0,86	12,75	82,10	11,16	71,86	11,92	76,75
		D	14,63	15,00	0,98	8,28	56,60	6,30	43,06	5,77	39,44
15	24,9	G	16,23	16,00	1,01	9,25	56,99	3,42	21,07	2,27	13,99
		D	7,73	14,70	0,53	4,78	61,84	3,98	51,49	3,59	46,44
17	23,5	G	12,45	15,10	0,82						
Moyenne			13,14	16,28	0,77	8,74	64,80	6,30	46,78	5,26	39,06
