# Supplementary material for: Beneficial effects of resistance training on both mild and severe mouse dystrophic muscle function as a preclinical option for Duchenne muscular dystrophy
Source: PLoS One. 2024 Mar 8;19(3):e0295700. doi: 10.1371/journal.pone.0295700 (PMC10923407; doi:10.1371/journal.pone.0295700)
Supplement: S4 File — Plan D2-mdx, Blots values (experiment 3). (PDF) [file pone.0295700.s005.pdf]

MHC-2a	D2-mdx								D2-mdx+OVL					
	1,59887	0,507438	0,458913	1,434779					1,01979354	2,281424	1,487458	1,866081	2,307453	
MyodD	D2-mdx								D2-mdx+OVL					
	0,551536	1,10142	1,471586	0,875458					1,511072	2,525345	2,667784	1,946262	1,904517	
utrophin	D2-mdx								D2-mdx+OVL					
		0,319441	1,229244	1,719425	0,73189				2,14302423	1,733425	1,949855	1,924633		1,584698
	D2-mdx								D2-mdx+OVL					
Akt	0,906253	1,258591	1,000719	0,834437					1,03772289	0,93578	1,053036	1,196324	1,549747	
pAkt	0,353574	1,477974	1,181372	0,98708					1,4971581	1,702388	1,660793	2,062513	2,269007	
pAkt/Akt	0,39731	1,19586	1,20219	1,20464					1,46921269	1,852607	1,606094	1,755683	1,490986	
	D2-mdx								D2-mdx+OVL					
CIII	1,159457	1,000803	0,874614	0,965127					1,23703538	1,319759	1,103728	0,883266	1,079955	
CIV	1,252821	0,801025	0,756272	1,189882					1,16280963	1,284034	1,166053	0,92602	0,98376	
CV	1,155069	1,014002	0,964785	0,866144					1,00876732	0,806077	0,997589	0,920338	0,968003	
gamma-actin	D2-mdx								D2-mdx+OVL					
	0,770445	1,252284	1,054655	0,922617					2,55461544	1,188537	2,030707	2,647797	2,36662	
desmin	D2-mdx								D2-mdx+OVL					
	1,039118	0,927201	0,755885	1,277795					1,10834348	1,761033	1,368159	1,410401		0,864854
