# Supplementary figures and images for: Beneficial effects of resistance training on both mild and severe mouse dystrophic muscle function as a preclinical option for Duchenne muscular dystrophy
Source: PLoS One. 2024 Mar 8;19(3):e0295700. doi: 10.1371/journal.pone.0295700 (PMC10923407; doi:10.1371/journal.pone.0295700)

# Utrophin

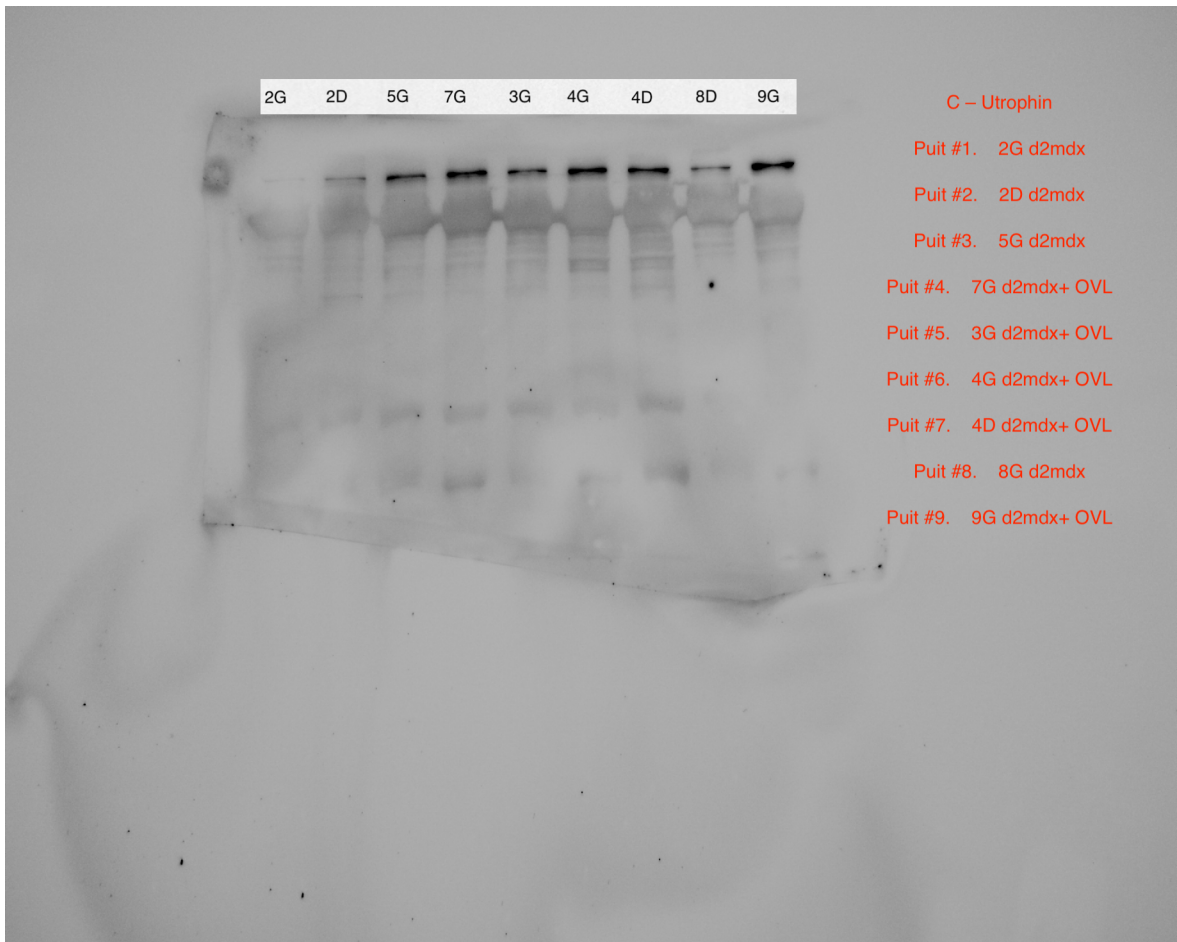

## HSP60

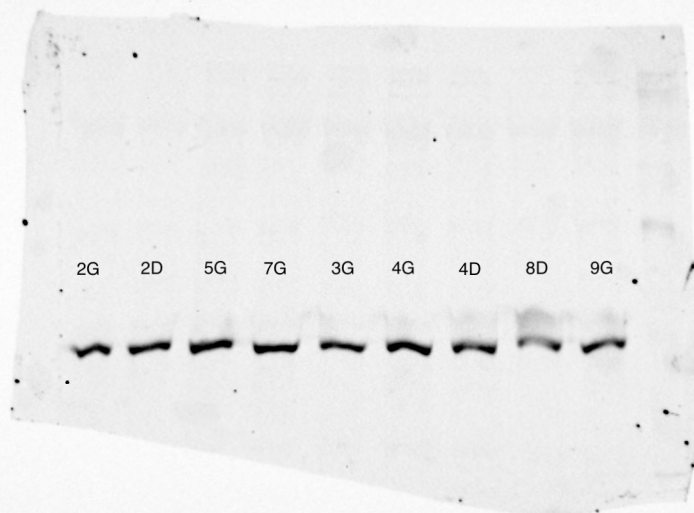

Supplement: S1 Raw image — Image of blot. (PDF) [file pone.0295700.s006.pdf]

gamma actin

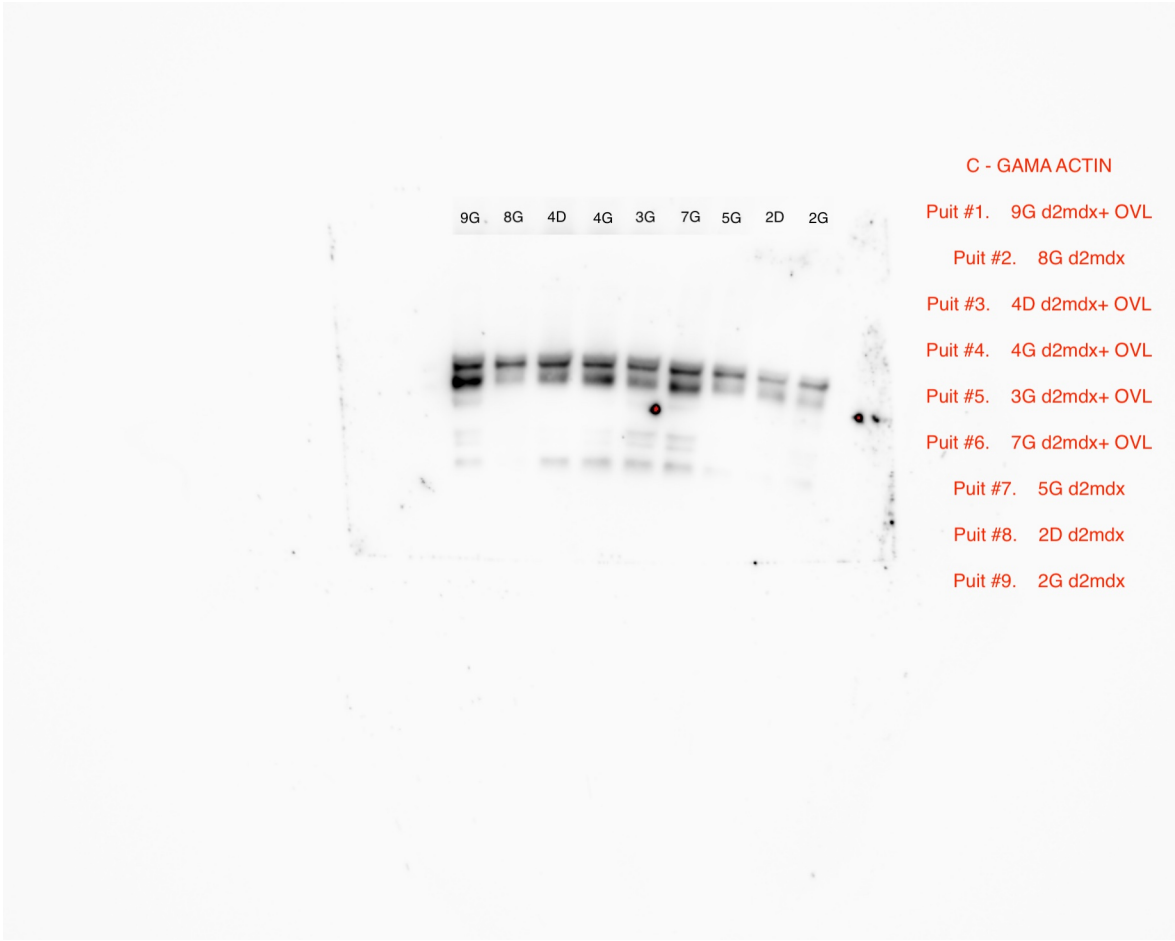

## HSP60

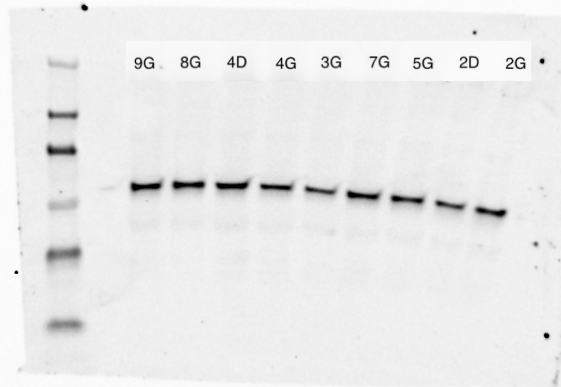

Supplement: S2 Raw image — Image of blot. (PDF) [file pone.0295700.s007.pdf]

desmin

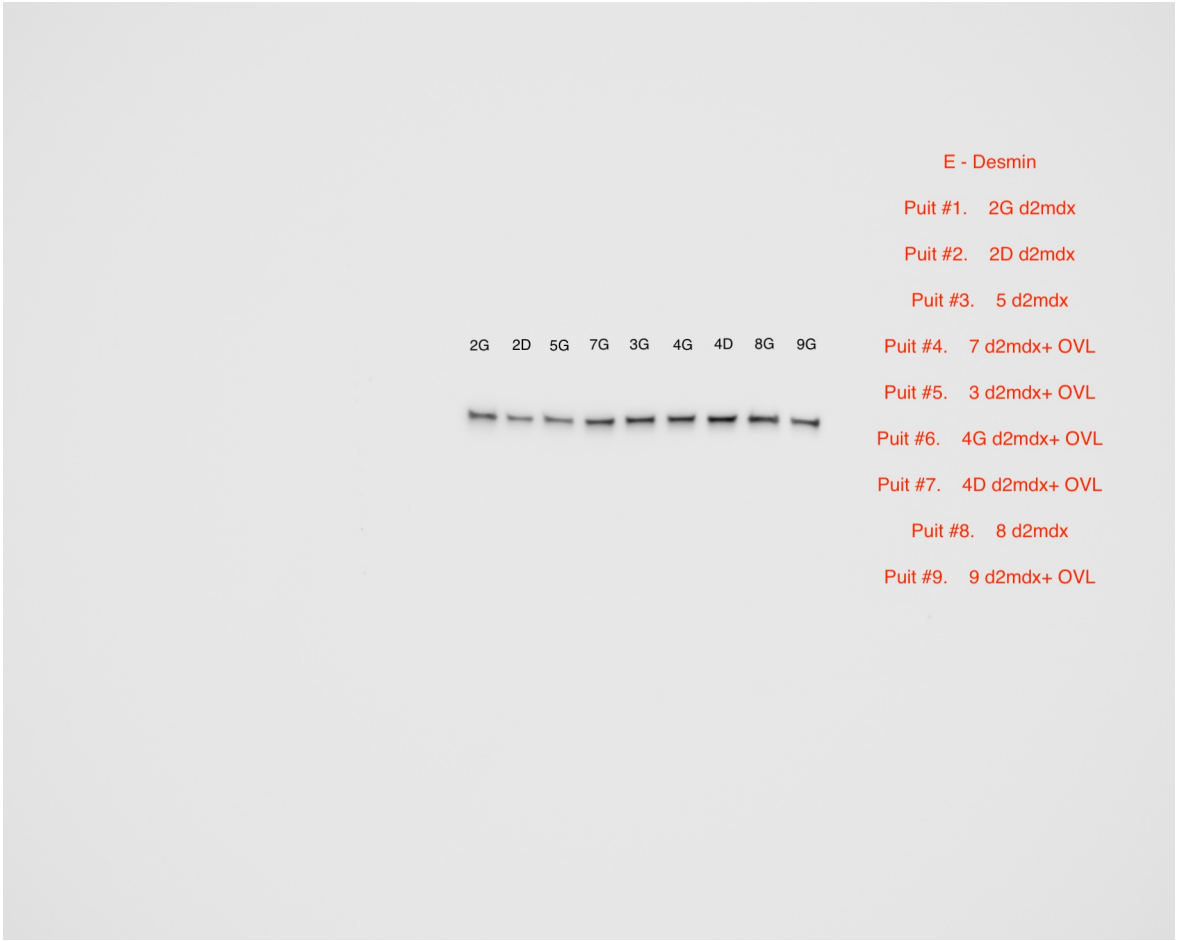

## Hsp60

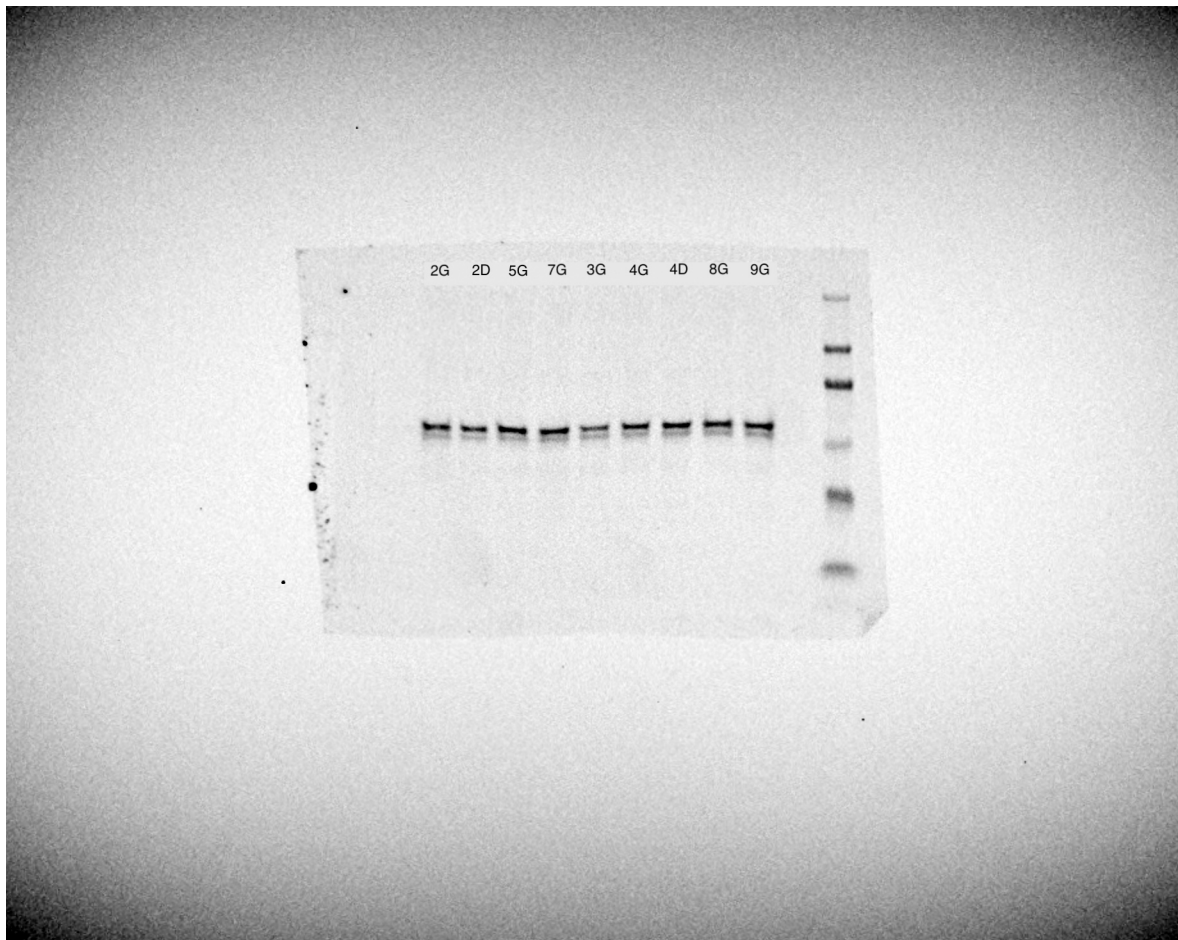

Supplement: S3 Raw image — Image of blot. (PDF) [file pone.0295700.s008.pdf]

## pAKT

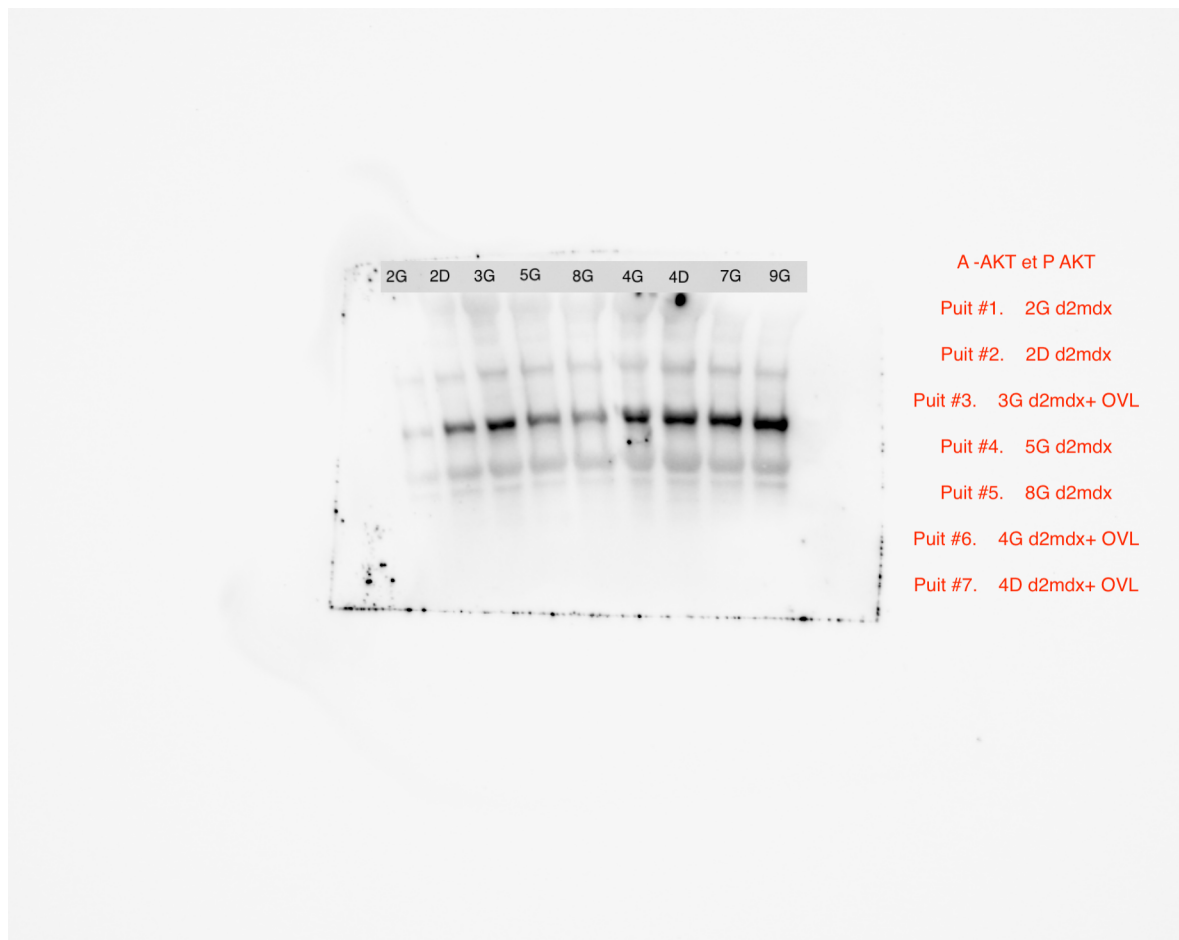

Akt

2G 2D 3G 5G 8G 4G 4D 7G 9G

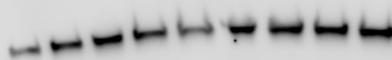

## HsP60

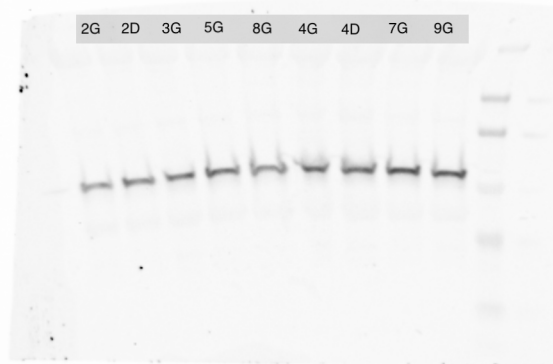

Supplement: S4 Raw image — Image of blot. (PDF) [file pone.0295700.s009.pdf]
