# Supplementary material for: Beneficial effects of resistance training on both mild and severe mouse dystrophic muscle function as a preclinical option for Duchenne muscular dystrophy
Source: PLoS One. 2024 Mar 8;19(3):e0295700. doi: 10.1371/journal.pone.0295700 (PMC10923407; doi:10.1371/journal.pone.0295700)

Respiratory complexes

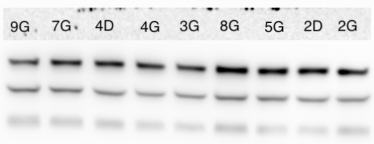

F – Respiratory chain

Line/Puit #1. 9G d2mdx+ OVL

Puit #2. 7G d2mdx+ OVL

Puit #3. 4D d2mdx+ OVL

Puit #4. 4G d2mdx+ OVL

Puit #5. 3G d2mdx+ OVL

Puit #6. 8G d2mdx

Puit #7. 5G d2mdx

Puit #8. 2D d2mdx

Puit #9. 2G d2mdx

HSP60

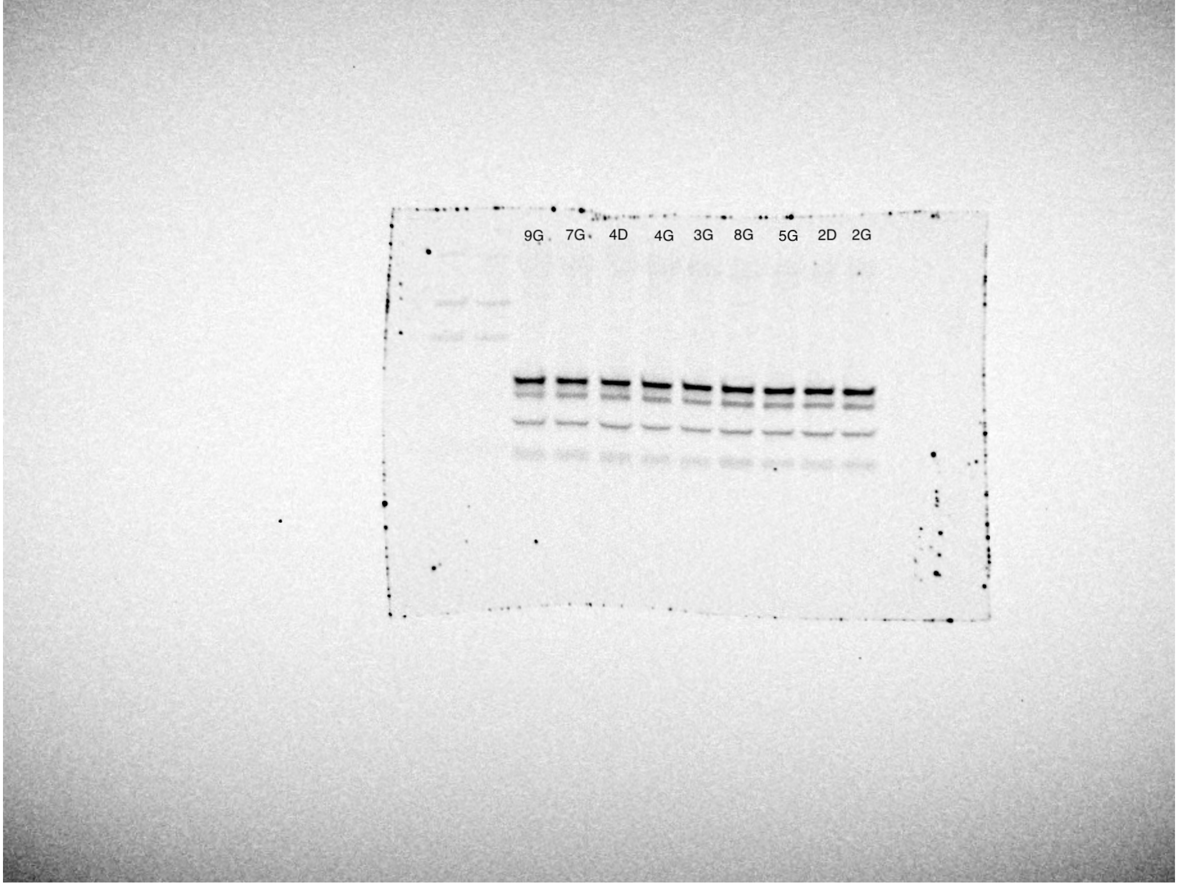

Supplement: S6 Raw image — Image of blot. (PDF) [file pone.0295700.s011.pdf]
